# The frontiers of medical negligence and diagnosis: an interview-based analysis

**DOI:** 10.1093/medlaw/fwad009

**Published:** 2023-05-30

**Authors:** Annie Mackley, Kathleen Liddell, Jeffrey M Skopek, Isabelle Le Gallez, Zoë Fritz

**Affiliations:** Faculty of Law, University of Cambridge, Cambridge, UK; Faculty of Law, University of Cambridge, Cambridge, UK; Faculty of Law, University of Cambridge, Cambridge, UK; Formerly Faculty of Law and THIS Institute, University of Cambridge, Cambridge, UK; Cambridge University Hospitals, THIS Institute and School of Clinical Medicine, University of Cambridge, Cambridge, UK

**Keywords:** Bolam, Diagnosis, Montgomery, Patients, Test, Treatment

## Abstract

While errors in medical diagnosis are common and often litigated, the different dimensions of diagnosis—formation, communication, recording—have received much less legal attention. When the process of diagnosis is differentiated in this way, new and contentious legal questions emerge that challenge the appropriateness of the *Bolam/Bolitho* standard. To explore these challenges, we interviewed 31 solicitors and barristers and asked them: (i) whether *Montgomery* should apply to information about alternative diagnoses; and (ii) whether the *Bolam/Bolitho* standard should be rejected in ‘pure diagnosis’ cases. Our qualitative analysis of the interviews sheds light not only on the two questions posed, but also on three cross-cutting themes. First, *Bolam/Bolitho* is criticised on two grounds that are often conflated: its paternalism for patients and its deference to medical professionals. Second, adopting different standards for different aspects of treatment and diagnosis may be justified in principle, but it can sometimes be difficult or not make sense in practice. Third, new conceptions of patients, doctors, and courts are being articulated in terms of rights or responsibilities over risks. In mapping these issues at the frontiers of medical negligence, this empirical study identifies potential pressure points for future legal developments.

## I. INTRODUCTION

While errors in medical diagnosis are common and have been the subject of much attention in the field of medical negligence,^1^ the different dimensions of diagnosis have only recently begun to be properly unbundled in law and medicine.^^2^^ As our prior work demonstrates, there are three distinct types of diagnostic acts that should be distinguished: the formation of the diagnosis, the communication of the diagnosis to the patient, and the recording of the diagnosis.^3^ Unbundled in this way, it becomes clear that each dimension raises important and complex legal questions that are worthy of distinct analysis. These questions challenge the assumption that all three dimensions of diagnosis should be governed by the *Bolam/Bolitho* standard (under which medical practice cannot be found negligent if it is accepted by a body of medical practitioners, unless the court finds the practice cannot withstand logical scrutiny).^4^ Given the importance of medical diagnosis, the enduring critiques of the *Bolam/Bolitho* standard, and the frequency of patient complaints about diagnosis, these questions constitute some of most important frontiers of medical negligence law.

This article is part of a broader project exploring medical diagnosis from clinical, legal, ethical, and anthropological perspectives. Amongst our previous papers is an article in this journal assessing the legal standards of care that apply to the formation, communication, and recording of diagnoses.^5^ When deciding whether a doctor has negligently breached the duty of care owed to a patient during medical diagnosis, what standard defines reasonable conduct for doctors? In that paper, we showed that case law is not settled. Typically, courts in England and Wales have applied the *Bolam/Bolitho* standard on the grounds that diagnostic decisions are grounded in clinical judgement. However, lower courts in *Muller v King’s College Hospital NHS Foundation Trust*^6^ and *Brady v Southend University Hospital NHS Foundation Trust*^7^ have invited senior courts to depart from *Bolam/Bolitho* in situations they call ‘pure diagnostic’ cases. Furthermore, the Supreme Court decisions in *Montgomery v Lanarkshire*^8^ and *Darnley v Croydon Health Services NHS Trust*^9^ cast doubt on the applicability of *Bolam/Bolitho* for, respectively, communication and recording of diagnoses. In analysing these issues, our prior work adopted a standard doctrinal methodology,^10^ but we identified the need for additional empirical research,^11^ which this article provides. Thus, we recommend that this article be read in conjunction with our 2022 publication in this journal, which provides more detail and analysis of the background case law.^12^

One of the central insights of our prior work is that different standards might apply to the different acts in the diagnostic context. If so, this would parallel the ways that different standards apply to different acts in the treatment context, where *Bolam/Bolitho* applies to the performance of the treatment itself, while *Montgomery* applies to the communication of the treatment risks. Clarifying the standards that apply in the diagnostic context is essential, as these standards must be incorporated in medical education, clinical guidelines, and patient care. This clarification is also relevant to legal proceedings and could lead to different results in some circumstances.^13^

In our prior work, however, we were not able to reach a clear answer for two of our questions: (i) whether *Montgomery* should apply to information about alternative diagnoses and (ii) whether *Bolam/Bolitho* should not apply in cases of ‘pure diagnosis’. The limited reported case law on these issues, along with the fact that the vast majority of medical negligence complaints are resolved without going to court, created blind spots here. To answer these questions, we interviewed barristers and solicitors who could provide insight into how these types of diagnosis cases are being resolved—insights based on empirical data that could not be obtained from other sources. We asked for their views on the standards that apply to the formation and communication of diagnosis, and on the direction in which the law should develop.

In this article, we present and analyse the results of these interviews, which shed light not only on the two questions posed but also on three cross-cutting themes. First, *Bolam/Bolitho* is criticised on two grounds that are often conflated: its paternalism and its deference to the medical profession. Second, adopting different standards for different aspects of treatment and diagnosis may be justified in principle, but there might be circumstances in which this is difficult or does not make sense in practice. Third, new conceptions of patients, doctors, and courts are being articulated in terms of rights or responsibilities over risks. In mapping these issues at the frontiers of medical negligence, this article identifies pressure points for potentially significant legal developments in the near future.

## II. METHODOLOGY

An initial list of potential participants was created by reviewing medical negligence judgments, which list the parties’ barristers and instructing firms of solicitors. Further potential participants were added through snowballing. Between 11 December 2020 and 25 February 21, one of us (ILG) invited 134 medical negligence lawyers to participate in the study via email. This email contained a link to the study’s THISCOVERY webpage which contained a participant information sheet, consent form and booking platform where the participant could opt for a telephone call or an online interview.

A semi-structured interview guide was generated from case law analysis in previous studies and two workshops held in Cambridge: the first (in July 2019) had 20 participants drawn from across law, medicine, philosophy, sociology, and anthropology; the second (in January 2020) focused on practicing and academic doctors and lawyers. At the second workshop, participants were asked to read the prepared reviews of case law and literature, in order to identify areas that needed further exploration. Through this process, we decided to focus the interviews on two questions that our research had identified as being highly contested in case law and literature: whether *Montgomery* should apply to the communication of information about alternative diagnoses and whether *Bolam/Bolitho* should not apply in cases of ‘pure diagnosis’.^14^ We refer to these as the ‘core questions’.

In total, 31 interviews with barristers and solicitors were undertaken by ILG over a period of three months in 2021. Table 1 provides more details about the participants, who were a mix of claimant-focused, defendant-focused and mixed practice lawyers whose experience ranged from six to 40+ years.

**Table 1.** Participants’ background and experience

Year of experience, years	Barristers	Solicitors
0–9	2	0
10–19	5	3
20–29	8	5
30–39	4	2
40+	2	0
Total	21	10

These interviews were recorded and transcribed verbatim by a professional transcription service. The transcripts were anonymised by giving each participant a numerical designation and indicating whether they were a solicitor or a barrister.

The interviews were analysed in NVivo Pro12 by ILG, ZF and AM using the Framework approach.^15^ Framework is an approach to qualitative analysis designed for applied (often policy-related) research, intended to enable systematic, transparent analysis of empirical datasets, often by teams, towards identified practical objectives.^16^ After reading the first transcripts, initially identified themes were used to generate a coding framework, which was discussed with an external collaborator (GM); transcripts were coded by three researchers with legal or medical expertise (ZF, AM and ILG) and consolidated and iterated in team discussions. Themes generated during the analysis process were discussed and validated by wider members of the research team (JS and KL). Data collection and analysis were concurrent, with early findings directing further enquiries in interviews. Illustrative quotes of the emerging themes identified are to be found in Table 2.

**Table 2.** Emerging themes and illustrative quotations

Theme	Illustrative quotations
Dissatisfaction with *Bolam/Bolitho* in communicating diagnostic uncertainty	‘It should be confined to the dustbin of history. It’s not acceptable in the modern world.’ (BAR 58786)
	‘I think *Montgomery* is nibbling away at *Bolam* quietly.’ (BAR 59716)
	‘I think *Montgomery* has opened the door in the sense that we now know very clearly that the *Bolam* test doesn’t apply to one area, which is the provision of information and advice to patients. And the fact that the court has been very clear in saying it doesn’t apply there, I think does sort of open the possibility that it may not apply elsewhere.’ (SOL 59953)
	The body of doctors test in *Bolam* has got a kind of momentum that, in my view, it doesn’t really deserve.’ (BAR 59823)

The emphasis on autonomy in communicating diagnoses	‘I think it is because when you are talking through with your patient what you are planning to do next, which they do now. I mean, they didn't, you know, 30/40 years ago. You just got told, this is what we’re going to do. They wouldn’t even tell you what the diagnosis… the differential diagnosis was. Or even what the working diagnosis was. And I think those days have gone. And that is partly due to *Montgomery* but it think it’s a, sort of, rather wider change in medical practice.’ (BAR 59663)
	‘The *Montgomery* mindset is wider than just that. The *Montgomery* mindset is, what can I do to empower my patient and make my patient my equal so that everything that I do is in dialogue with my patient? And I give them the information that they need to be an equal partner in how we go about addressing whatever is that brought them through the door?’ (BAR 59949)
	‘I think the point of *Montgomery* and some of the cases after is that the … it’s moving away from the paternalism approach to medicine. And I think the extension to that must be that the patient ought to be informed of not only if there is a diagnosis what the diagnosis is; but of the … well, to an extent the differential diagnosis. I don’t think it would be reasonable to expect a doctor to list off every possible differential diagnosis the patient might have.’ (SOL 59657)
	‘If paternalism is dead and *Montgomery* kind of killed paternalism then it seems to me that patients should be fully informed, should be part of the process and informed of not just the reasonable treatment options but the reasonable differential diagnosis.’ (SOL 60968)
	‘*Montgomery* obviously a shift towards focusing on autonomy and the right of the patient to sort of be involved in and make decisions about their own care, and I think it’s fair to say that’s gone wider than just consent issues and those sorts of cases’, ‘*Montgomery* is not all-encompassing in that sense, but it does indicate a broader shift towards a focus on the rights and I was going to say interests of the patient but I suppose what I mean more is the rights of the patient to be involved in their own care and their own diagnosis, which is a significant shift away from *Bolam*.’ (BAR 60618)
Dissatisfaction with *Bolam/Bolitho* in pure diagnosis	‘The reason I think it’s spot on is that, there's a lovely irony about this, which is that the *Bolam* test, the *Bolam* body of doctors test, includes the reference to the word, logical. Because it's the *Bolitho* element of that test is that it has to withstand the logical analysis of risk and benefits. And the reason that I'm in agreement with the Judge in *Muller* is that, there is a complete illogicality, ironically, trying to apply that test to say the interpretation of a slide, as I think it was in *Muller*. Because either the abnormality is there, or it isn't.’ (BAR 61460)
	‘My own personal view is that, applying the *Bolam* body of doctors test to scenarios where it just clearly is illogical to apply it, we should call that out and just say, look, this is not right.’ (BAR 59823)
	‘There haven’t been that many decided cases on what you might call pure diagnosis, and the obvious legal test would be reasonable care: has the doctor exercised reasonable care and skill in making the diagnosis? It seems that where the law has got to is a complete muddle and we have to apply the *Bolam* test, which makes no sense, to diagnosis whatsoever. But the way the court gets round the illogicality of the *Bolam* test is to apply the *Bolitho* and say, well, we’ll be suspicious, exercise a high degree of suspicion about the defence. A reasonable body of doctors would have made this mistake? You have to ask the question: would it be logical to reach that diagnosis?’ (SOL 59953)
	‘I mean I do notice that when people write, a lot of lawyers writing about cases make comments about *Bolam* is the test to breach a QC. And I always look at that and raise my eyebrow and think, well, you know, it is in some cases but it’s not in all cases. And we need to be a bit more sophisticated.’ (SOL 59953)
	‘It strikes me that *Bolam* should still apply though. I mean, you could get it wrong but it depends on why you’ve got it wrong and what was going on, and if an expert would say, well, yes, you got it wrong but actually it was very difficult to get it right and a responsible body would have made the same mistake, then yes, I don’t see why *Bolam* wouldn’t apply in that case.’ (BAR 60618)
	‘You know, it doesn’t really make sense to me to say *Bolam* doesn’t apply because it’s a binary choice. In a way, a lot of medical decisions are binary choices, right? You know, you either treat or you don’t treat or you diagnose or don’t diagnose that thing. So yes, I do struggle to understand the reasoning there.’ (BAR 60618)
	‘I can see the difference but you’ve still ultimately got professionals having to exercise professional judgment around the diagnosis and around the interpretation of the histopathology. And so, whilst you can retrospectively say that, you know, objectively yes it was, or it wasn’t this, when you’re judging the standard that’s been given by the histopathologist, reviewing that slide, then surely you’ve got to apply the same standards as you would for a treatment case. Because they need to do what a responsible body of professional histopathologists would do.’ (SOL 60340)
	‘Personally, I don’t think it (deference) should have some role to play. I think, unfortunately, it does have some role to play because the courts have said it does, but I don’t really think it adds to anything. So the issue is, what is there on the slide to be seen?’ (SOL 59953)
	‘I also think that there could be scope for the judge to decide the particular findings of fact. It looked like this, as a matter of fact, not it was an area of uncertainty. It could have been one thing or it could have been the other. But it was actually condition A, not condition B, when you look at it. It was malignant cell. I would take the *Bolam* at face value. Just because another expert says it was okay, frankly I don’t think it’s going to pass muster.’ (SOL 60969)
	‘There are certainly cases where you think, why did they miss it? You know, barn door. But there are also cases where there’s genuine argument, you count up the number of abnormal nuclei in the field you’re looking at or the shape of the cells, or whatever it may be.’ (BAR 60224)

Treatment/diagnosis distinction in pure diagnosis cases	‘Whenever I look at a case, I’m very careful to work out, is this a *Bolam* body of doctors case, is this a pure diagnosis case of reasonable skill and care, is it a histology case, is it an interpretation case, is it a *Montgomery* case?’ (BAR 59823)
	‘It’s interesting if you’re saying that radiology can either be right or wrong, there’s either a lesion there or not. I think there is still interpretation in that.’ (SOL 60506)
	‘So my view is that actually pure diagnosis is sort of when … I think it’s difficult because I don’t think everything is always that cut and dried.’ (SOL 61613)
	‘Yeah, so he’s kind of right in saying that there is something different with diagnosis versus treatment, because usually treatment is being done on the basis of a diagnostic determination that already exists. And the treatment is a series of options for which you can consent or not consent to. There is a difference in them, but there is more of a blurring than maybe that might indicate.’ (BAR 59769)
	‘I don’t think there is ever a clear demarcation between the two, because actually the whole process is one of interaction between clinician and patient both ways, an information exchange. And so actually, I’m not sure that the kind of purity of the division is something that could be sustained.’ (BAR 59716)
	‘I can’t really see any advantages at the moment, I mean, certainly not in my work, to having a different test applying to diagnosis as to treatment. They do go very hand in hand. They go hand in hand in a doctor’s mind as well, so if ultimately this is a test of the doctor’s decision-making and his ability to provide reasonable advice to his patient then I can’t really see why you’d divide the two.’ (SOL 59178)

Treatment/diagnosis distinction in communicating diagnostic uncertainty	‘It seems to me that by definition when you’re thinking of treatment options you have also got to be thinking of differential diagnosis, so I don’t know whether your brain can untangle it in the way that the lawyers are trying to get them to do it.’ (BAR 59820)
	‘It’s almost always going to be the case if there is a dispute about diagnosis failing to consider, but it’s going to be about failing to consider a serious differential and, therefore, failing to treat it.’ (BAR 59928)
	‘It seems, to me, and I’ve probably said this already, that *Montgomery* only really strays into this where there are decisions to be made.’ (BAR 59928)
	‘I think it’s when treatment starts to be involved with that that Montgomery sort of comes more to the front of my mind.’ (BAR 60618)
	‘I think the test will have to be, and this is where I think *Montgomery* is aligned, it’ll have to be where a diagnosis is unclear and treatments—diagnostic treatments, not cures but exploratory treatments—are required.’ (SOL 59657)
	‘I do think we’re still in *Bolam* territory and I don't think that *Montgomery* particularly plays out, unless you’re looking at consent to diagnostic processes or a treatment pathway.’ (SOL 60340)
	‘If there is uncertainty, that predicates that there is a risk in pursuing a particular line of treatment based on that diagnosis and that must come within the parameters of *Montgomery*, in my view.’ (BAR 58982)
	'On one level you can start breaking that down into treatment versus diagnosis or *Bolam* v *Montgomery*, but on another level it’s actually just well, what does the patient need to know to be informed about the next steps? And I think if you take a much more holistic, pragmatic approach you don’t get bogged down in these tiny distinctions.’ (BAR 59820)

Risk management in communicating diagnoses	‘And there are risks involved in having that, but there’s a one per cent risk, if we don’t do this, that we will miss the thing that’s inside of you, that is ultimately going to be very, very serious. I think, in that situation, of course, the clinician must tell the patient what the something serious is, because there are … there’s a decision to be made there, there are risks and benefits on either side and that is precisely within *Montgomery*.’ (BAR 59928)
	‘Most cases, the patient has a right to know and is entitled to know the ups and the downs and the risks of … the concerns that a doctor has.’ (BAR 59749)
	‘It’s very hard to see why patient autonomy shouldn’t apply to the question of which possible diagnoses should be investigated? You know, my body, my choice. It’s very odd. If I’m entitled to say what should be done to my body, why am I not entitled to say what should be looked into? Doctor, I don’t want you to instigate the possibility of prostate cancer, because, whatever it may be, my dad had a horrible treatment which I don’t want to undergo, or it brings up memories that I don’t want to deal with. Why shouldn’t I have that option?’ (BAR 60224)
	‘*Montgomery* isn’t actually … just about informed consent, it’s probably more accurately about risk and disclosure of the risk, so to the extent that decisions had to be made about risk or I suppose decisions have to be made more broadly about how a person discharges their right to personal autonomy, then questions of a diagnosis could be relevant to that context.’ (BAR 60225)

This study (IRAS ID: 265331) was approved by the East of England—Essex Research Ethics Committee.

## III. RESULTS AND ANALYSIS

### A. Should *Montgomery* apply to information about alternative diagnoses?

#### 1. Context

Following the Supreme Court decision in *Montgomery*, doctors must ensure that their patients are aware of any ‘material risks’ involved in any recommended treatments, and materiality is defined by a two prong test: ‘The test of materiality is whether, in the circumstances of the particular case, *a reasonable person in the patient's position* would be likely to attach significance to the risk, or the doctor is or should reasonably be aware that *the particular patient* would be likely to attach significance to it.’^17^ In adopting this patient-led standard, and rejecting a ‘profession-led’ standard,^18^ the Supreme Court explained that the latter would sanction differences in practice that were not attributable to different interpretations of medical science, but merely ‘divergent attitudes among doctors as to the degree of respect owed to their patients’.^19^

In the literature, *Montgomery* is widely described as a significant move towards a healthcare model based on a partnership between patient and doctor, elevating patient autonomy and shifting away from the traditionally paternalistic medical professional-led approach.^20^ Rather than simply disclose what a logical body of doctors would consider it necessary to disclose, the treating doctor must consider the patient’s perspective, objectively and subjectively. However, the full scope and contours of this patient-based duty remain unsettled.^21^

There are two related dimensions of uncertainty that are relevant to our enquiry. First, *Montgomery* states that doctors must inform their patients of ‘reasonable alternative or variant treatments’, but does not explain whether this reasonableness is defined by a patient-led or a profession-led standard. Second, because *Montgomery* was a case about risks of proposed treatments, the Court did not explain whether doctors must also inform their patients of other types of related risks or information. For these reasons, one key unresolved question is whether a doctor might be required to disclose reasonable alternative diagnoses, and if so, whether a doctor-led or patient-led standard would apply.

An extension of *Montgomery* to some diagnostic matters is not hard to imagine, given that information about alternative diagnoses and related risks can have the same value for patients as information about treatment risks. For example, it can be valuable in making autonomous decisions about one’s healthcare, and it can also be helpful in avoiding harm (for example, where fatal or serious diagnoses are on the list of differential diagnosis, or where a patient needs to respond to changing or worsening symptoms).^22^ However, it is conceivable that extending *Montgomery* in this way could create additional time pressures on doctors, encourage defensive medicine, or increase litigation.^23^

Questions about the extension of *Montgomery* to the communication of diagnostic information have not yet been addressed by the courts of England and Wales,^24^ though equivalent questions have been addressed in other jurisdictions, including Scotland,^25^ Singapore,^26^ and the USA.^27^ One of the main aims of our interviews, therefore, was to explore whether lawyers in England and Wales thought that *Montgomery* could require the disclosure of diagnostic information, and whether they were, in fact, bringing these types of claims.

#### 2. Interview results

Most interviewees suggested that *Montgomery* should extend to the disclosure of alternative diagnoses on the ground that the principle of patient autonomy demands that patients be fully informed of diagnostic options.^28^ They thought that *Montgomery* required doctors to involve patients in their own care, including in diagnosis. As one solicitor put it,

*‘Patients should be fully informed, … part of the process, and informed of not just the reasonable treatment options but the reasonable differential diagnosis*’.^29^

Extending *Montgomery* to differential diagnosis was seen as an application of the move away from paternalistic healthcare to a partnership between doctor and patient; one interviewee summarised this view by saying that

*‘[Doctors should ask:] what can I do to empower my patient and make my patient my equal so that everything I do is in dialogue with my patient?’*^30^

This approach enables patients to assess risks and benefits in conjunction with their doctors and make decisions based on risk-benefit analyses that align with their personal values and priorities. A number of interviewees pointed out that, just as there are risks and benefits with treatments, there are similar risks and benefits associated with differential diagnosis, since

*if there is uncertainty, that predicates that there is a risk in pursuing a particular line of treatment based on that diagnosis*.^31^

Interviewees suggested that the management of these risks was a responsibility to be shared between patients and medical professionals, rather than left to one or the other. They therefore thought that the disclosure of differential diagnoses fell squarely within the parameters of *Montgomery*.

Interviewees also provided a number of examples of cases from their own practices which illustrated that failure to disclose diagnostic uncertainty can materially affect a patient’s agency in making medical decisions. In one case, a patient with high-blood pressure, obesity, and a history of smoking presented to A&E with neurological symptoms. It was unclear whether they were suffering from migraine or whether they had experienced a Transient Ischaemic Attack (TIA), sometimes described as a ‘mini-stroke’. The patient was ultimately diagnosed with migraine but was told that they needed to change their lifestyle in order to avoid suffering a stroke. They later suffered a stroke and argued that, had they been informed that they *might* have had a TIA—and that this was the reason for the lifestyle advice—they would have taken the advice more seriously lost weight and not suffered a stroke at all.

The interviewee thought that this type of case fitted better within the parameters of *Montgomery*, rather than *Bolam/Bolitho—*that the patient’s complaint related not to treatment received or an incorrect diagnosis, but rather to the failure to fully disclose uncertainty over her diagnosis which had deprived them of informed decision-making agency as to their lifestyle.^32^ Harm might have been avoided had the patient been informed of the seriousness of her differential diagnoses. The interviewee thought that both the avoidance of harm and support for patient autonomy were key reasons for imposing a *Montgomery* duty in relation to the disclosure of diagnosis.^33^

In another example, an ultrasound of a pregnant woman revealed that the foetus had clubfoot. In most cases, clubfoot can be easily corrected but it can also be an indicator of a congenital syndrome known as arthrogryposis, which can lead to profound physical disability. In this case, the doctors chose not to inform the woman that arthrogryposis was on the list of differential diagnoses because the risk was small and they felt that ‘no mother would possibly want to have a termination for clubfoot’.^34^ However, the woman claimed that she personally would have asked to terminate the pregnancy if she had been informed of the risk of a serious disability like arthrogryposis, however small. The failure to fully inform her of alternative diagnoses had deprived her of the opportunity to make decisions about her pregnancy in accordance with her personal priorities.

Despite agreeing that, in principle, *Montgomery* should apply to the disclosure of diagnostic uncertainty, some interviewees raised concerns about differentiating the standards that define negligent diagnostic communication and negligent treatment (which is governed by the profession-led standard of *Bolam/Bolitho*). They raised two types of concerns. First, it could be difficult to draw a line between diagnosis and treatment. In their view, both formed part of a continuous medical encounter, where disclosure of diagnostic uncertainty has an inevitable effect on the treatment pursued by a patient. As one interviewee said,

*I don’t know whether your brain can untangle it in the way that the lawyers are trying to get them to do it*.^35^

Second, some interviewees questioned the *purpose* of trying to draw this line. They thought that in most cases, diagnostic uncertainty was important precisely *because* of its effect on a patient’s future treatment. Failure to disclose diagnostic uncertainty could result in the patient pursuing an unsatisfactory treatment pathway. As one interviewee said,

*It seems to me that, by definition, when you are thinking of treatment options you have also got to be thinking of differential diagnosis*.^36^

For this reason, they thought that a doctor who failed to disclose diagnostic uncertainty could be challenged under an ordinary application of *Montgomery* for failing to disclose reasonable alternative or variant treatments, or under *Bolam/Bolitho* for failing to recommend appropriate treatment. Furthermore, given the need for a claimant to prove damage, a claimant in such a case would almost inevitably point to harm arising from a *treatment* decision. Some interviewees thought that it was easier to characterise this sort of fact pattern as a negligent treatment case, bypassing the need to differentiate the relevant standard.

### B. Should *Bolam/Bolitho* not apply in ‘pure diagnosis’ cases?

#### 1. Context

There has long been a debate about the extent to which courts should defer to the medical profession in judging cases of medical negligence, and while it is generally agreed that a profession-led standard should be used, it is important to recognise that not all profession-led standards are equally deferential.^37^ For example, some common law jurisdictions adopt a standard that requires doctors to exercise reasonable skill and care as defined by their profession as a whole—and as determined by a judge or jury after hearing expert evidence. In England and Wales, by contrast, a doctor need only follow a practice that is accepted by some members of the profession under *Bolam*, and when there is a disagreement between experts about what a reasonable doctor would do, the judge is not authorised to decide which is right.^38^ Rather, under *Bolitho*, the judge is only authorised to reject a body of medical opinion ‘if, in a rare case, it can be demonstrated that the professional opinion is not capable of withstanding logical analysis’.^39^ Thus, although *Bolitho* limits *Bolam’s* deference to the medical profession, the *Bolam/Bolitho* approach adopted in England and Wales is still significantly more deferential than that in other common law jurisdictions.

This deference has given rise to the question of whether there are cases of misdiagnosis in which *Bolam/Bolitho* should not apply. For example, it has been argued that a different standard should apply in ‘pure diagnosis’ cases that involve only a ‘diagnosis of the condition … with no decision made or advice given about treatment or further diagnostic procedures’,^40^ and where there cannot be two ‘right’ or ‘respectable’ answers.^41^ Medical experts at the centre of such cases might include histopathologists, radiologists, sonographers, cytoscreeners, and genetic laboratories.

While arguments to treat such cases differently were rejected by the Court of Appeal in *Penney v East Kent Health Authority*,^42^ Kerr J implicitly invited higher courts to revisit this question in *Muller v King’s College Hospital NHS Foundation Trust.*^43^ In that case, Kerr J stated *obiter* that the expert’s diagnosis in the case was based on a histopathological slide analysis that was straightforwardly right or wrong (in this case, wrong) and thus did not involve the exercise of medical judgement that justified deference under the *Bolam/Bolitho* approach.^44^ However, he explained that he was nevertheless required to follow the *Bolam/Bolitho* approach, and he found negligence under what he described as a ‘liberal invocation’ of the *Bolitho* exception.^45^

Kerr J’s critique of *Bolam/Bolitho* was also advanced by Deputy Judge Lewis QC in *Brady v Southend University Hospital NHS Trust*.^46^ In this case, he drew a distinction between ‘treatment cases’, where there are ‘choices and options available and risks and benefits that need to be considered’, and ‘pure diagnosis’ cases, where there is ‘there is no weighing of risks against benefits, and no decision to treat or not to treat’.^47^ Lewis J stated that in the latter, there is ‘limited scope for any genuine difference of medical opinion’ and that ‘a diagnosis based upon a scan is usually either right or wrong’.^48^ For this reason, he suggested that *Bolam/Bolitho* should not apply to such cases. However, like Kerr J, he concluded that he was bound by *Penney*,^49^ and that, in any event, the facts of *Brady* did not fall in the pure diagnosis category.^50^

As *obiter dicta* from respected judges can lead to appeals and, potentially, legal developments, we decided to ask the interviewees for their views on the decision in *Muller*.

#### 2. Interview results

There was a strong division of opinion between those interviewees who supported *Muller’s* diagnosis/treatment distinction and those who did not.

Those in favour of Kerr J’s reasoning supported it on the basis that they thought the deferential approach to the opinion of a body of medical experts in *Bolam/Bolitho* was inappropriate when it came to questions of fact. One interviewee suggested that this was ‘*a complete illogicality*’ because the *Bolam/Bolitho* test was designed to apply where there is a range of options (for example in relation to treatment) for which doctors must weigh risks and benefits and could reasonably come to different conclusions.^51^ The rationale for the *Bolam/Bolitho* test did not apply to pure diagnosis questions, where the doctor was either right or wrong. Some interviewees suggested that courts could reduce *Bolam*-style deference with the *Bolitho* exception in pure diagnosis cases, by asking whether it was logical to reach that diagnosis, but they concluded that this was unsatisfactory. As one interviewee stated, ‘*we need to be a bit more sophisticated*’ by properly identifying cases in which *Bolam/Bolitho* is—and is not—appropriate.^52^

Part of the interviewees’ support for Kerr J’s reasoning in *Muller* seemed to be driven by a broader dissatisfaction with the deferential approach taken in *Bolam/Bolitho.* They thought that doctors should not have so much authority over the legal standards that apply to them. Particularly in ‘pure diagnosis’ cases, medical experts should not have the last word on the applicable standard where the medical issues appear to turn on questions of fact, such as whether a diagnosis was correct or not. Rather, these cases were better resolved by applying the test of reasonable skill and care that applies elsewhere in the law of negligence, under which the judge—taking into account expert evidence—decides whether an expert has acted reasonably. They suggested that *Bolitho* did not always solve the problem of unjustified deference and that the *Bolam/Bolitho* approach had been applied too broadly.^53^ As one interviewee stated,

*the body of doctors’ test in Bolam has kind of got a momentum that, in my view, it doesn’t really deserve*.^54^

On the other hand, a large proportion of interviewees opposed Kerr J’s reasoning in *Muller* on the basis that a clear line cannot be easily drawn between ‘pure diagnosis’ and ‘pure treatment’ cases. They argued that the vast majority of the former cases are actually exercises in interpreting data which can lead to different views. Although most interviewees acknowledged that there were some cases in which there was eventually a clear right answer, they thought that, in most cases, there was room for ‘genuine argument’ over how to interpret the data at the time of the diagnosis.^55^ One barrister believed that this kind of interpretation called for clinical expertise:

*Even in something like the interpretation of a scan, there is room for more than one view, and it’s not necessarily negligent to prefer one view over the other*.^56^

This view was echoed by a number of interviewees who pointed out that the process of interpretation requires doctors to make judgement calls, informed by their clinical knowledge and experience, as to the most likely diagnosis and the appropriate next steps. One interviewee in particular noted that it was very difficult to separate out this interpretive exercise from other elements of the medical encounter that involve skill and expertise, since making a diagnosis is part of a continuous process of interaction between doctor and patient.^57^ Interviewees who opposed Kerr J’s reasoning concluded that the degree of clinical expertise involved in making what Kerr J termed ‘pure diagnosis’ made it appropriate for assessment by a responsible body of medical opinion under *Bolam/Bolitho*.

### C. Cross cutting themes at the frontiers of clinical negligence

Our interviews not only shed light on the two questions that we posed, but also revealed a set of three common themes that underly these frontiers of clinical negligence.

#### 1. Criticism of Bolam/Bolitho

Dissatisfaction with the *Bolam/Bolitho* standard was a common thread in the interviews. Many interviewees were critical of its widespread application across very different types of cases in the past,^58^ and they were sceptical about whether it should apply to both the communication and formation of diagnoses. They often favoured different standards of care for different aspects of clinical practice.

It is important to recognise, however, that their reasons for rejecting *Bolam/Bolitho* differed across the two questions. When asked what standard should apply to the communication of diagnoses, they criticised *Bolam/Bolitho* on the grounds that it allows a paternalistic approach to the practice of medicine. This concern was grounded in respect for patient autonomy. By contrast, when asked about what standard should apply in pure diagnosis cases, they criticised *Bolam/Bolitho* on the grounds that it is overly deferential to the medical profession. This critique was grounded in rule of law concerns about the proper role of the judiciary.

The two different critiques also focus on different domains of decision-making. The first focuses on *Bolam/Bolitho’*s impact on the relationship between doctors and patients, advocating a greater role for patients in *medical* decision-making. The second focuses on *Bolam/Bolitho’*s effect on the relationship between the medical profession and the court, advocating a reduced role for doctors in *legal* decision-making.

Interviewees did not always differentiate between these two sets of reasons for rejecting *Bolam/Bolitho*, but it is important to avoid conflating them when the law develops at these two frontiers. While rejections of paternalism and rejections of deference can support the same changes in the law, as in *Montgomery*, this will not always be the case.

#### 2. Differentiating treatment and diagnosis

Both of our questions assumed that a clear line can be drawn between treatment and diagnosis, but this is a relatively new exercise in the law of clinical negligence, which has typically lumped them together.^59^ For example, the *Bolitho* exception applies to ‘cases of diagnosis and treatment’,^60^ and *Hunter v Hanley* (the Scottish equivalent of *Bolam*) states that ‘in the realm of *diagnosis and treatment* there is ample scope for genuine difference of opinion’.^61^ It is therefore not surprising that the desirability and feasibility of differentiating treatment and diagnosis was a second cross-cutting theme.

Two points are worth flagging here.

First, many interviewees no longer assumed, as the law long has, that diagnosis and treatment should be governed by a single standard. Rather, they carefully considered whether and how treatment and diagnosis should be disentangled, and the standards that should apply to each. For example, some interviewees stated that there cannot be a genuine difference of opinion in pure diagnosis cases, contrary to the *Hunter v Hanley* quotation above. The importance of recognising different standards was aptly summarised by a barrister who said that in every case, they asked themselves:

*Is this a Bolam body of doctors case? Is this a pure diagnosis of reasonable skill and care? Is it a histology case? Is it an interpretation case? It is a Montgomery case?*^62^

This demonstrates a substantial departure from previous approaches that elided the difference between diagnosis and treatment under a single standard.

Second, some thought that differentiating standards was justified in principle, but that there were circumstances in which it was difficult or did not make sense in practice. For example, some interviewees thought that in relation to both the core questions, treatment and diagnosis could be intimately connected, making it difficult to apply different standards to each. They also questioned the practical value of drawing the distinction, given that many common fact patterns could be characterised as either negligent treatment or negligent diagnosis; for example, a failure to communicate diagnostic uncertainty that leads to incorrect and harmful treatment. They suggested that in these circumstances, disaggregating the standard along a treatment–diagnosis boundary added unnecessary complexity and did not help to delineate the scope of the *Montgomery* and *Bolam/Bolitho* standards.

Thus, it seems that a tension and challenge for medical law may lie ahead. The trend among practitioners, as well as in reported cases, is to move away from the application of a single standard to all types of cases. But, as interviewees’ discussion of the treatment/diagnosis distinction reveals, it can sometimes be difficult to disaggregate and differentiate standards on grounds that are coherent and significant in practice.

#### 3. Rights and responsibilities over risk

A third cross-cutting theme in our interviews was the articulation of new rights and responsibilities in terms of the risks inherent in pursuing medical treatment. This can be seen in both the medical and legal domains.

In the medical domain, this was identifiable in answers to the question of whether *Montgomery* should apply to the communication of diagnostic uncertainty. Here, many interviewees commented on how the role of responding to risk should be distributed among the different actors in the diagnostic process. Most suggested that at least some rights or responsibilities should lie with the patient, necessitating the disclosure of at least some diagnostic uncertainty.

In the legal domain, this theme can be seen in answers to the question of whether *Bolam/Bolitho* should apply to pure diagnosis cases. Here, some interviewees argued that the applicability of *Bolam/Bolitho* should be defined and limited on the basis of whether a doctor is making a decision based on the weighing of risks. They suggested that if a doctor is weighing risks, such that there is not an objectively correct decision, the reasonableness of the decision should be determined by *Bolam/Bolitho* (at least while this remains the general standard for those exercising professional skill)*.* However, in pure diagnosis cases, where they saw no weighing of risks, but rather a decision that was either correct or incorrect, they concluded that reasonableness should be determined by a judge based on expert testimony.

Thus, across both domains, interviewees were thinking in terms of risk management when defining the relative rights and responsibilities of patients, doctors, and judges.

## IV. CONCLUSION

Our interviews focused on two questions at the frontiers of medical negligence law that emerged from our prior work: (i) whether *Montgomery* should apply to the communication of alternative diagnoses, and (ii) whether *Bolam/Bolitho* should not apply in ‘pure diagnosis’ cases. Most interviewees concluded that *Montgomery* should apply to communicating alternative diagnoses, as they thought that patient autonomy requires patients to be informed and involved in the diagnostic process, especially where diagnostic uncertainty affects a patient’s decisions about treatment. Opinions on cases of pure diagnosis, however, were more divided. Some interviewees thought that *Bolam/Bolitho* should not apply on the grounds that these cases do not involve the weighing of risks in choosing between different options that can all be correct. Others thought that most diagnoses (including those that are termed ‘pure diagnosis’) involve interpretation and the possibility for reasonable disagreement, thus warranting the type of deference provided by *Bolam/Bolitho.*

While the interviews did not settle our core questions to the extent that we had hoped, they confirmed that these issues are at the frontiers of medical negligence where we may see legal developments in the near future, with advocates bringing these debates before the courts. We have previously argued that the different dimensions of medical diagnosis raise interesting and complex legal issues,^63^ and our interviews revealed that lawyers in the field are increasingly thinking about these issues and applying their views in practice (including when it comes to settling cases). Our interviews also provided a wealth of experience and empirical examples against which to assess the views we had explored in our body of work to date. Prevailing assumptions in the field, such as the widespread application of *Bolam/Bolitho* and the dominance of the medical practitioner in managing the diagnostic process, are being challenged. Other themes, such as the careful disaggregation of different parts of the medical encounter, are also coming to the fore. These indicate a future direction of travel for the law of medical negligence, through which we hope the legal and practical complexities highlighted by our core questions will be further unravelled and clarified.

## Footnotes

1 M Jones, *Medical Negligence* (5th edn, Sweet & Maxwell 2018) [4-10]–[4-50]; J Laing and others (eds), *Principles of Medical Law* (4th edn, OUP 2017) s 4.74.

2 K Liddell and others, ‘Differentiating Negligent Standards of Care in Diagnosis’ (2022) 30 Medical Law Review 33, <https://doi.org/10.1093/medlaw/fwab046>; M Khazen and others, ‘Anatomy of Diagnosis in a Clinical Encounter: How Clinicians Discuss Uncertainty with Patients’ (2022) 23 BMC Prim Care 153, doi: 10.1186/s12875-022-01767-y; National Academies of Sciences, Engineering, and Medicine, *Improving Diagnosis in Health Care*, Chapter 2: The Diagnostic Process (The National Academies Press 2015), <https://doi.org/10.17226/21794>.

3 Liddell and others (n 2).

4
*Bolam v Friern Hospital Management Committee* [1957] 1 WLR 582; *Bolitho v City & Hackney Health Authority* [1998] AC 232.

5 ibid.

6 [2017] EWHC 128 (QB).

7
[2020] EWHC 158 (QB).

8 [2015] UKSC 11.

9 [2018] UKSC 50.

10 We also conducted a systematic literature review to assess the impact of *Montgomery* in the six years since the Supreme Court’s landmark decision. See I Le Gallez and others, ‘*Montgomery*'s Legal and Practical Impact: A Systematic Review at 6 Years’ (2022) 28 Journal of Evaluation in Clinical Practice 690.

11 Liddell and others (n 2) 59; Le Gallez and others (n 10) 699.

12 Liddell and others (n 2).

13 ibid 52–54.

14 ibid 58.

15 J Ritchie and J Lewis (eds), *Qualitative Research Practice: A Guide for Social Science Students and Researchers* (Sage 2003).

16 ibid; J Ritchie and L Spencer, ‘Analyzing Qualitative Data’ in A Bryman and R Burgess (eds), *Qualitative Data Analysis for Applied Policy Research* (Routledge 1994).

17
*Montgomery* (n 8) [86].

18 The different types of standards and their origins is discussed in Liddell and others (n 2) 37–40.

19
*Montgomery* (n 8) [84].

20 This view is described and evaluated in Le Gallez and others (n 10) 692. Prior to *Montgomery*, the legal standard for disclosure of medical risk was unsettled. On one view, the majority in *Sidaway v Board of Governors of the Bethlam Royal Hospital* [1985] AC 871 considered the matter to fall within the scope of *Bolam*, subject to two qualifications. However, courts had departed from this view in various cases. *Montgomery* (n 8) [86].

21 Le Gallez and others (n 10) 6–7.

22 Liddell and others (n 2) 52.

23 Similar concerns have been repeatedly expressed about Montgomery’s impact on treatment practices: Le Gallez and others (n 10) 7–9.

24 Liddell and others (n 2) 49–52.

25
*Taylor v Dailly Health Centre and others* [2018] CSOH 91. In this case, the court held that *Montgomery* did not require the disclosure of diagnostic uncertainty on the grounds that there is a crucial distinction between: (a) considering investigatory or treatment options and (b) discussing a recommended treatment and possible alternatives with the patient. [40].

26
*Hii Chii Kok v Ooi Peng Jin London Lucien* [2017] SGCA 38. This decision introduced a modified *Montgomery* standard. It held that a doctor was required to disclose information which a reasonable patient in the patient’s position would wish to know (in line with *Montgomery*). Unlike the decision in *Montgomery* (which was silent on this point), the Singaporean Court expressly highlighted information pertaining to other possible diagnoses such as the degree of certainty, the reasons for the lack of certainty and whether more can be done to clarify the uncertainty. However, whether a doctor was negligent in failing to possess that information and in failing to disclose it remained to be assessed by reference to the *Bolam/Bolitho* standard. The decision is discussed in more detail in Liddell and others (n 2) 50–51. Since then, the Singapore Parliament enacted legislation responding to *Hii Chii Kok.* Section 37 of the Singaporean Civil Law Act now provides, in brief, that a doctor providing advice to a patient meets the standard if he acts in a manner accepted by a responsible body of logical medical opinion (seemingly reintroducing *Bolam/Bolitho*). It also provides, however, that medical opinion must require the provision of the information that a person in the same circumstances as the patient would reasonably require to make an informed decision, and information that ought to be known to be material to the patient in question based on questions asked by the patient or information gleaned from their medical records (seemingly retaining the elements of *Montgomery* which were adopted in *Hii Chii Kok*). The meaning and effects of this provision have yet to be determined. See generally, K Amirthalingam, ‘Upending the Medical Duty to Advise: Legislating the Standard in Singapore’ (2022) 22 *Medical Law International* 189.

27
*Jandre v Wisconsin Injured Patients & Families Compensation Fund* 813 NW2d 627 (Wis 2012). The Wisconsin Supreme Court held that a physician could have a duty to disclose information regarding diagnostic uncertainty under the ‘reasonable patient’ standard that was in place at the time. The decision and the legislation that followed is discussed in more detail in Liddell and others (n 2) 50–51.

28 They supported the decision in *Hi Chii Kok.*

29 SOL 60968.

30 BAR 59949.

31 BAR 58982.

32 BAR 60433.

33 See also Liddell and others (n 2) 52–54.

34 BAR 60244.

35 BAR 59820.

36 BAR 59820.

37 For a brief overview, see Liddell and others (n 2) 37–39.

38 Of course, some judges may nevertheless do so: R Mulheron, *Principles of Tort Law* (CUP 2020) 365–66.

39
*Bolitho v City and Hackney HA* [1998] AC 232 (HL), 243.

40
*Muller v King’s College Hospital NHS Foundation Trust* [2017] EWHC 128 (QB), [48], [62]. It seems that some have suggested that ‘pure diagnosis’ cases could also include situations where no further action is taken after the diagnosis; for example, the claimant’s lawyers in *Brady* suggested that it was a case of pure diagnosis. This was rejected by the judge. See *Brady* (n 7).

41 E Jackson, *Medical Law: Text, Cases and Materials* (5th edn, OUP 2019), 134 (citing *Muller* [75]).

42 [1999] EWCA Civ 3005.

43 [2017] EWHC 128 (QB).

44
*Muller* (n 40) [72].

45 ibid [97].

46
*Brady* (n 7).

47 ibid, at [23].

48 ibid.

49 ibid.

50 ibid, at [20]. The case involved the misdiagnosis of gastrointestinal infection. Unlike a ‘pure diagnosis’ case, the doctors made decisions on the basis of the diagnosis (for example, to treat with a basic course of IV and oral antibiotics, and to discharge home without urgent gastroscopy). [6]–[14]. Furthermore, the claimant’s argument was that the doctors should have performed a test to confirm a specific diagnosis. See Liddell and others (n 2) 41–44, and *Brady* (n 7) [20].

51 BAR 61460.

52 SOL 59953.

53 The extent to which the *Bolitho* exception reduces judicial deference is also questioned in literature. See generally, Mulheron (n 38) 369–75; M Brazier and J Miola, ‘Bye-bye Bolam: A Medical Litigation Revolution?’ (2000) 8 Medical Law Review 85, 88.

54 BAR 59823.

55 For example, if a child has encephalopathy when they are born, they also had it when they were a foetus at 20 weeks gestation. At the time of a prenatal scan, however, the scan would require interpretation and there might be room for reasonable disagreement about whether the fetus had encephalopathy. At birth, when the child is older and outside the womb, the condition is clearer.

56 BAR 61332.

57 BAR 59716.

58 This is not to say that it has been applied in all types of case. See generally Mulheron (n 38) 376–81. For example, *Bolam/Bolitho* does not apply in: disclosure of risk cases (where *Montgomery* now applies, and prior cases applied versions of a patient-led perspective); matters that the court considers to be questions of ‘fact’ (where the court’s view on the balance of probabilities is determinative); cases where the crucial issue does not involve matters of expert or clinical judgement (for example, providing administrative information about waiting times, as in *Darnley v Croydon Health Services NHS Trust* [2019] AC 831); and cases where there is no body of professional opinion.

59 Liddell and others (n 2) 34.

60
*Bolitho v City & Hackney Health Authority* [1998] AC 232 (HL), 243.

61
*Hunter v Hanley* [1955] SC 200, 204 (emphasis added).

62 BAR 59823.

63 Liddell and others (n 2).

